# Modelling Food Substitution Using the Ofcom Nutrient Profiling Model on Population Intakes from the Canadian Community Health Survey–Nutrition 2015

**DOI:** 10.3390/nu16121874

**Published:** 2024-06-14

**Authors:** Qiuyu (Julia) Chen, Misa Gillis, Jodi T. Bernstein, Adelia Jacobs, Conor L. Morrison, Mahsa Jessri

**Affiliations:** 1 Food, Nutrition and Health Program, Faculty of Land and Food Systems, The University of British Columbia, Vancouver, BC V6T 1Z4, Canada; 2 Faculty of Medicine, University of British Columbia, Vancouver, BC V6T 1Z3, Canada; 3 Department of Statistics, Faculty of Science, The University of British Columbia, Vancouver, BC V6T 1Z4, Canada; 4 Centre for Health Services and Policy Research (CHSPR) and Health Services and Policy (HSP), Faculty of Medicine, The University of British Columbia, Vancouver, BC V6T 1Z3, Canada

**Keywords:** nutrient profiling, dietary intakes, Canada, food supply, public health, policy

## Abstract

This study aimed to model how substituting foods consumed by Canadians for alternatives with more favourable nutrient profiling (NP) scores would impact dietary intakes. The Ofcom NP system, developed to help the UK Office of Communication differentiate foods that can be advertised to children, was applied to foods consumed by Canadians aged 2 years and older in the 2015 Canadian Community Health Survey (CCHS) (*n* = 19,447). Foods were substituted for similar options from the Euromonitor branded food composition database (Scenario 1) or from the primarily aggregated food profiles in the CCHS survey food composition database (Scenario 2) with either the most favourable (optimistic; 1A and 2A) or a more favourable Ofcom score (realistic; 1B and 2B). Mean intakes of Ofcom scores, calories, saturated fat, sugars, and sodium from these scenarios were compared to baseline. Only 2.9% of foods consumed had a similar Euromonitor option with a lower Ofcom score. Scenarios 1A, 1B, and 2A had lower Ofcom scores, calorie, sodium, saturated fat, and sugar intakes compared to baseline. Scenario 2B had lower levels of all outcome measures, except for an increase in calories compared to baseline. Selection of foods with more favourable NP scores has the potential to decrease the Canadian intake of nutrients of concern.

## 1. Introduction

Globally, the burden of chronic disease is staggering [1]. In Canada, over two-thirds of adults have a body mass index in the overweight or obese categories [2], 25% and 11% of adults live with hypertension and diabetes, respectively [3], and cardiovascular disease is the second-leading cause of adult death in the country [4]. A major and modifiable risk factor for these diseases is poor nutrition, specifically diets containing excess energy, saturated fats, sugars, and sodium [5]. Decreased intake of these nutrients of concern has been associated with several health benefits [6]. Sodium intake < 2 g daily has been shown to reduce blood pressure, and lower daily sodium intake is correlated with reduced risk of stroke and coronary artery disease [7], decreased sugar intake is associated with lower body weight [8], and a diet lower in saturated fat has been linked to lower incidence of combined cardiovascular events [9,10].

Studies have shown that Canadians consume more saturated fats, free sugars, and about twice as much sodium as recommended [11,12,13,14,15,16,17]. To promote improved nutrition and reduce chronic disease risk, Health Canada has developed a Healthy Eating Strategy, which encompasses several food and nutrition policies [18]. Changes that are part of the strategy include a new food guide [19]; an update to the nutrition facts table and ingredients list on packaged foods [20]; an update to the voluntary sodium reduction targets for processed foods [21]; the introduction of mandatory front-of-package (FOP) nutrition labelling on foods high in saturated fats, sugars, or sodium [22]; restricting the advertising of similar “high-in” foods to children (e.g., sugar-sweetened beverages) [23]; and strengthening access to nutritious foods through Nutrition North Canada [18].

For policies related to food reformulation, marketing, and labelling, evaluating the healthfulness of foods is an essential step. There are numerous nutrient profiling (NP) systems that score foods according to their nutrient content to aid with nutrition decision-making and comparison between products [24]. NP models underpin nutrition policies such as restrictions on the marketing of foods to children, FOP labelling, standards for institutions (e.g., schools), subsidies or taxes, regulation of nutrition or health claims, and nutritional surveillance [24]. The use of NP models can impact population intakes and public health outcomes through the encouragement of reformulation and by helping guide consumer food choices [25,26].

One such system is the Ofcom system developed in the United Kingdom for the purpose of determining which foods could be marketed to children [27]. The Ofcom system considers the amount of calories, sugars, saturated fats, sodium, fibre, protein, and percentage of fruits, vegetables, and nuts to determine a continuous score with thresholds for categorizing foods and beverages as “healthier” and therefore permitted to be marketed to children [27]. The Ofcom model has been extensively validated in the literature and also served as the basis for the development of several additional NP models [24]. When adopted into a dietary index, the Ofcom model has demonstrated predictive validity for body weight [28], metabolic syndrome [29], cardiovascular disease [30], and cancer [31]. With the goal of informing nutrition policy in Canada, our study models how substitutions for healthier food choices defined by the Ofcom system could impact nutrient and calorie intakes at a population level in Canada.

## 2. Materials and Methods

**Figure 1:**
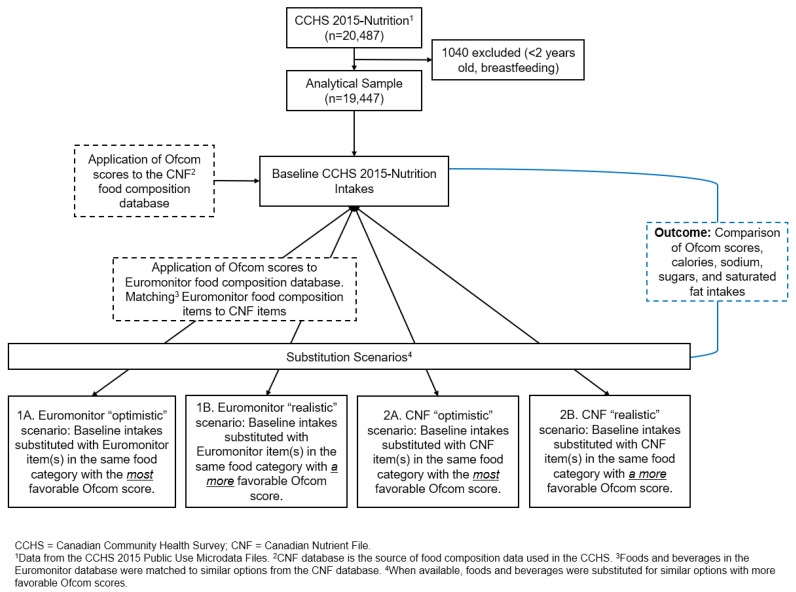
Process flow diagram and overview of study design.

This study modelled four scenarios in which the dietary intakes of Canadians were altered by “replacing” foods and beverages (foods) in the diet with foods that have better NP scores when such foods were available. Briefly, using the Ofcom NP model and dietary data collected from the 2015 Canadian Community Health Survey (CCHS)–Nutrition, we modelled scenarios that substituted foods reported as consumed in the CCHS 2015 survey with similar foods from the same food category that had lower Ofcom scores. Substituted foods were obtained from two different food composition databases (Euromonitor and the Canadian Nutrient File (CNF)) and under the assumptions of both realistic and optimistic substitution scenarios (Figure 1).

### 2.1. Databases Used

Dietary intakes of Canadians were calculated from the 2015 CCHS Public Use Microdata Files (PUMFs). The CCHS–Nutrition 2015 is a nationally representative, cross-sectional, population-level health survey with a nutrition focus conducted by Statistics Canada in partnership with Health Canada. The survey provides detailed information on dietary consumption and collects data on demographic, anthropometric, and socio-economic variables [32]. Participants surveyed lived in private dwellings in the 10 Canadian provinces and did not include members of the Canadian forces, residents of the Northwest Territories, Yukon, Iqaluit, Indigenous reserves or settlements, institutions, or some remote areas. The CCHS–Nutrition 2015 includes a 24 h dietary recall for 20,487 people aged 1 and above, with a second 24 h recall conducted for a subset of the sample [32]. The current analysis excluded children aged less than 2 years and people who were breastfeeding. The final sample included 19,447 individuals. Data collection for this survey was granted ethical approval by Statistics Canada and collected under the authority of the Statistics Act of Canada. Informed consent for participation was obtained by Statistics Canada from all subjects involved in the study. A complete description of the CCHS study design can be found elsewhere [32].

The CNF is the national food composition database of Canada and is the source of food composition data applied to the 24 h dietary recalls in the CCHS survey [33]. The CNF food composition database contains information primarily on aggregated food products (nutrition information is a compilation from multiple brands of similar food products). If reported foods had missing nutrient data, these nutrient data were calculated using the corresponding information in the CNF database and the weight of the food consumed as reported in the CCHS. If the CNF also excluded nutrient information for a specific item, then the median nutrient value of foods from the same food category (Bureau of Nutritional Sciences (BNS) food categories included in the CNF) was used to replace the missing information.

To garner data on a range of possible outcomes and to perform realistic food substitutions for the primarily generic food compositions in the CNF, the Euromonitor International database of available individually branded products [34] was also used as a source of nutritional values for substitute foods in the modelled scenarios. Euromonitor is a market research firm that collects data on consumer purchases of various goods in 54 different countries. Their database of brand-name packaged foods available in Canada includes information on the nutrient content and market share of each product. To find appropriate substitutions, Euromonitor foods were matched to the most similar food in the CNF. The methodology for this matching has been previously published [35]. Briefly, match options were first generated using fuzzy matching and an algorithm based on thresholds of maximum nutritional differences [35]. If a suggested match was deemed nutritionally appropriate, it was selected. If no appropriate matches were suggested by the algorithm, the Euromonitor food was either matched manually to a CNF food or deemed unmatchable. Manual matching was performed independently by two or more team members with dietetics qualifications. A total of 27 out of 1179 Euromonitor products were deemed unmatchable. In total, 512 of 6392 (8.0%) foods reported as consumed in the CCHS–Nutrition 2015 were matched to a similar food in the Euromonitor database [35].

### 2.2. Calculation of Ofcom Scores

The Ofcom NP system was applied to all foods in the CNF and Euromonitor databases. In the Ofcom model, points are assigned for increasing amounts of energy, saturated fats, sugars, and sodium, while points are subtracted for increasing proportions of fruits/vegetables/nuts and amounts of fibre and protein [27]. Therefore, foods with lower Ofcom scores have more favourable nutrient profiles. Although Ofcom scoring takes into account the percentage of fruits, vegetables, and nuts in a food, ingredient lists were not available in the CNF and Euromonitor databases, and only the nutrient content of each food was known. As such, assumptions had to be made to calculate a complete Ofcom score, decided through consensus among team members with dietetic qualifications. Therefore, Ofcom minimum, low, medium, and high scores were calculated for each food to represent four possible percentages of fruit, vegetables, and nuts. For some food products, the percentage assigned was at one end of the extreme based on the item name (e.g., apples would be >80%, the highest point level, and cow’s milk would be ≤40%, the lowest point level), but for items with less obvious compositions (e.g., a granola bar with fruit and nuts), the middling percentage options (1 point for >40% and 2 points for >60%) were averaged to generate an Ofcom score to be used in the analysis.

Ofcom scores, which were applied to all foods, were then used to calculate a dietary Ofcom score for the dietary intakes from the CCHS–Nutrition 2015. Dietary Ofcom scores are the weighted mean of the scores of all foods reported in each individual’s recall, calculated according to Equations (1) and (2), where the weight of food consumed was taken from the 24 h recall, and the reference amount of food was taken from the CNF.

(1) $$\mathrm{Portion}=\frac{\mathrm{Weight}\;\mathrm{of}\;\mathrm{food}\;\mathrm{consumed}\;\left(g\right)}{\mathrm{Reference}\;\mathrm{amount}\;\mathrm{of}\;\mathrm{food}\;\left(g\right)}$$

(2) $$\mathrm{Dietary}\;\mathrm{Ofcom}\;\mathrm{score}=\frac{\Sigma \left(\mathrm{Ofcom}\;\mathrm{score}\;\times \;\mathrm{Portion}\right)}{\Sigma \mathrm{Portion}}$$

### 2.3. Substitution Scenarios

Foods reported as consumed in the CCHS–Nutrition 2015 survey that had a similar option from the same BNS food category with a lower Ofcom score were substituted to create alternative 24 h recalls that could be used to determine usual intakes. Four different substitution scenarios were modelled. Scenarios varied to exhibit alternatives in choice in terms of selecting substitutes based on market availability and nutritional composition. The Euromonitor and CNF databases were each used to obtain substitute foods (Scenarios 1 and 2, respectively), and within those options, either the most favourable Ofcom score (“Optimistic”, using the food(s) with the *lowest* score in the same food category) (Scenarios 1A and 2A) or a lower Ofcom score (“Realistic”, using the food(s) with a *lower* score in the same food category) (Scenarios 1B and 2B) were chosen. Any foods reported as consumed for which there was no appropriate substitute with a lower Ofcom score were left as is.

Specifically for Scenario 1, if there were multiple Euromonitor substitutes with the same Ofcom score, a new food ID was created that represented the average nutrition information for the lowest-scoring option (Scenario 1A) or the nutrition information weighted by market share data for multiple lower-scoring options (Scenario 1B). Because of the nature of the Euromonitor database, Scenario 1 was limited to substitutions for packaged food items.

For Scenario 2, if there were multiple CNF matches with the same *lowest* Ofcom score (Scenario 2A), the lower-calorie food was chosen. For three BNS food categories (“01C—Cereal Grains and Flours”, “51A—Tea”, and “51B—Coffee”), a substitute was chosen that did not have the lowest Ofcom score in the category because the lowest-scoring food was found not to be representative of the category and resulted in significantly higher energy and sugar intake in the population. For example, “Coffee substitute, cereal grain beverage, powder” had the lowest Ofcom score (score of −3) in the category “51B—Coffee”, however, it contained 3.61 calories per gram, while most options in this category had fewer than 1 calorie per gram. This resulted in a large caloric increase when modelled at the population level. Instead, the substitute chosen was “Coffee, brewed, prepared with water,” which had the next-lowest Ofcom score of 0 as well as zero calories and sugar. Scenario 2B used a substitute from the same food category with the *next-lowest* Ofcom score. If there were multiple foods with the *next-lowest* Ofcom score, the lower-calorie food was chosen. Scenario 2 included substitutions of unpackaged and packaged foods.

Once a match was found, the nutrient composition and Ofcom scores replaced the food reported as consumed in the CCHS 24 h recall. Finding matches with lower Ofcom scores and creating new food IDs were carried out using Microsoft Excel and R.

### 2.4. Analysis

Analyses were weighted to represent the Canadian population, and a balanced repeated replication method with 500 bootstrap weights was used to estimate variance. Descriptive analyses were applied to the first 24 h recall data, available for all respondents, using PROC SURVEYMEANS. Mean dietary Ofcom scores and intake of calories, saturated fats, sugars, and sodium were calculated for the baseline CCHS–Nutrition 2015 dietary intakes and for each substitution scenario. Mean values for the substitution scenarios were compared to the baseline scenarios using paired *t*-tests. A subset analysis was conducted for Scenarios 1A and 1B, examining nutrient intake and Ofcom score for only the subset of foods that could be substituted with a Euromonitor food match (8.0% of reported foods in the CCHS had a Euromonitor match, and 2.9% had a match with a lower Ofcom score). Statistical significance was defined by a *p* value of <0.05. SAS software (version 9.4; SAS Institute Inc., Cary, NC, USA) was used.

## 3. Results

The final sample included 19,447 Canadians, comprising 9312 (47.9%) males and 10,135 (52.1%) females. This included 5729 (29.5%) individuals under the age of 19 and 13,718 (70.5%) individuals aged 19 and above. This sample had a baseline mean ± SEM reported intake of 1805.6 ± 12.5 calories, 22.8 ± 0.4 g of saturated fat, 2710.5 ± 30.7 mg of sodium, and 90.8 ± 0.9 g of total sugars (Table 1). Substituting foods with lower Ofcom scores from the Euromonitor and CNF databases resulted in significantly lower Ofcom scores, saturated fat, sodium, and total sugar intakes for all scenarios compared to baseline (Table 1). Calories were lower for Scenarios 1A, 1B, and 2A, but increased for scenario 2B. When examining only the subset of foods that were substituted with a Euromonitor match, the mean Ofcom scores, calories, saturated fats, sodium, and total sugars were significantly lower than baseline (Table 2).

## 4. Discussion

This study modelled four substitution scenarios in which the nutritional composition of products with more favourable NP scores replaced those of foods and beverages reported as consumed by Canadians in the CCHS–Nutrition 2015. All scenarios modelled represented those that could feasibly be made given the existence of items available in the Canadian marketplace. All substitutions were made within the same food category, limiting the requirement for major changes to consumers’ purchasing or dietary behaviours.

Examination of scenarios substituting items with lower Ofcom scores showed a reduction in mean daily calories, sodium, saturated fats, and sugars. Although not statistically compared, Scenarios 1A and 1B appeared to result in similar nutrient and Ofcom scores, suggesting that consumer selection of any substitution, even if it does not have the best NP score available, would confer comparable nutritional benefits. Observed changes were modest for Scenario 1, so the clinical significance of these substitutions may be minimal. Taking sodium as an example, although the substitution resulted in lower intakes, total consumption for Scenario 1 remained above the 2300 mg daily maximum recommended by Health Canada [12]. Prior research has demonstrated that a reduction in sodium intake to 2300 mg/day, albeit from a higher starting baseline (3250–3450 mg/day), resulted in significant decreases in blood pressure [36,37]. Future studies can examine how the modelled reductions in nutrient and calorie intakes from the present work are related to health outcomes. The modest difference from the baseline for Scenario 1 may also be due to the small number of favourable substitutions from the Euromonitor database (2.9%) and that the Euromonitor database includes only packaged foods, thus leaving many fresh or minimally processed foods reported in the CCHS–Nutrition 2015 without a substitution option. However, the variation in Ofcom scores for whole and unprocessed foods may be minimal as they tend to be less flexible to modification (e.g., banana). The limited availability of lower-scoring products in the Euromonitor database perhaps also indicates that Canadians are already choosing items from a food category with more favourable scores or that there is minimal range in Ofcom scores within a food category. As expected, in the subgroup analysis, limited to items consumed with a Euromonitor substitution, the relative decreases in nutrients of concern were more pronounced. Therefore, a larger selection of lower-Ofcom-scoring options available may increase the potential impact.

The scenarios with CNF substitutions differed from the Euromonitor scenarios, with especially pronounced decreases in the optimistic scenario (2A). As expected for a more realistic scenario, reductions for Scenario 2B were much more modest. Of note is that Scenario 2B saw a large increase in daily calories from baseline, while calories decreased for Scenario 2A. The manual substitutions performed for three food categories in Scenario 2A to account for large mean increases in daily calories were not conducted for Scenario 2B, perhaps explaining this discrepancy. Because the population intake of beverages from the categories “51A—Tea” and “51B—Coffee”, which were subject to manual substitutions for Scenario 2A, is high, small caloric increases per gram of coffee or tea amount to large dietary increases. As such, Ofcom scores may not be the best way to choose beverages in these categories, as coffees and teas generally have little nutritional value, but the addition of “positive” nutrients (e.g., fibre, protein) can increase the Ofcom score or offset the negative components of the food (e.g., sugars) while also increasing calorie content. Instead, selecting options lower in calories and sugars may be preferred for beverage categories, especially considering beverages have been found to contribute to 12.6% of Canadians’ total sugar intake [13].

Prior research, including modelling scenarios, has examined how the use of various NP models can guide intakes through food choices or reformulation and the impact this could have on health [38,39,40,41,42]. For instance, Kuar et al. found that the use of NP models to regulate health claims on foods may result in shifts towards less healthy diets as health claims tend to be on foods with better nutritional profiles and restricting the use of the health claims may make it more difficult for consumers to identify these foods. On the other hand, if the foods with health claims were all reformulated to meet the NP criteria required to carry a claim, this could result in positive dietary changes. Another modelling study that used a similar concept to the present work compared baseline intakes from a 2003 Dutch food consumption survey in young adults to substitution scenarios in which all foods not compliant with the Choices Programme NP system were replaced [42]. Similar to the present work, that study showed substitutions would shift population intakes towards dietary intake goals [42]. New Zealand provides an example of significant food reformulation by manufacturers in response to food labelling criteria, which resulted in the exclusion of approximately 33 tonnes of salt from foods in a one-year period [39]. This corresponded to an average 61%, 26%, and 11% reduction in sodium in breakfast cereals, breads, and margarine, respectively [39]. In the Canadian context, Emrich et al. found that if traffic light FOP labelling were applied in Canada and consumers chose foods without a red warning label, caloric intake would decrease by 5%, saturated fat by 14%, and sodium by 6% [38].

It should also be noted that this study focussed purely on the substitution of foods for similar products with better nutrient composition. While this is an important component in improving Canadians’ diets, aligning diet composition with public health recommendations is crucial. A varied diet focussed on vegetables and fruit, whole grains, and protein foods with minimal consumption of highly processed products is endorsed by Canada’s food guide [19]. Strategies to promote further adoption of these guidelines will be needed in parallel with other policies. For instance, Canada’s Healthy Eating Strategy includes the use of NP systems for front-of-pack labelling and the proposed restrictions on marketing to children [18,23].

This work has several limitations. Firstly, the modelling relied on assumptions about which foods would be used as substitutes. It was assumed that consumers would respond to NP systems by increasing consumption of foods with better scores and reducing consumption of those with less favourable scores. This assumption is supported by research showing that consumers are more likely to choose foods without “high in” labels identifying nutrients of concern [43,44,45] and that manufacturers reformulate products to achieve a healthier nutrient composition in response to NP policies [46,47,48,49]. However, other factors that influence food intake like food price, income, social environment, convenience, taste, and emotional state were not considered [50]. To address these uncertainties, we modelled several scenarios to explore possible variations that could be expected should consumers choose items with lower NP scores. The modelled substitutions, representing ideal scenarios, may have overestimated the nutrient differences that would be seen under real-world conditions. Overestimation is especially likely for Scenarios 2A and 2B, where all foods with appropriate substitutions were replaced, while only 2.9% of foods could be matched to a healthier alternative in Scenarios 1A and 1B. Overall, a reasonable expected change may fall somewhere between the outcomes of Scenarios 1 and 2. Our study included only one day of recalls, which allowed us to compare descriptive statistics of the baseline intakes with the modelled substitution scenarios but not to determine usual intakes, which take into account day-to-day variability. As with other dietary surveys using 24 h recalls, the data are limited by the recall bias accompanying such methods. To counteract recall bias, the automated multiple-pass method was used when collecting dietary data for the CCHS [51]. This work did not exclude pregnant people as the PUMFs do not include pregnancy as a variable as it is considered sensitive data. A major strength of this work is the use of food substitutions that represent real-world options available to consumers.

## 5. Conclusions

The findings of this study demonstrate the potential role of NP systems and nutrition policy in effecting positive change. High baseline population intake of saturated fats, sodium, and sugars decreased when foods were replaced with favourably scored alternatives according to the Ofcom NP system. Governmental action on proposed policies is necessary for these modelled benefits to be realized in an effort to improve Canadians’ diets and chronic disease risk.

## Figures and Tables

**Table 1:** Ofcom scores and nutrient intakes reported in the Canadian Community Health Survey–Nutrition (CCHS–Nutrition) 2015 PUMFs for baseline data and substitution scenarios (*n* = 19,447).

	Dietary Ofcom Score	Daily Calories, kcal	Daily Saturated Fat, g	Daily Sodium, mg	Daily Total Sugars, g
Baseline data	5.2 ± 0.1 [5.0, 5.5]	1805.6 ± 12.5 [1781.1, 1830.1]	22.8 ± 0.4 [22.1, 23.6]	2710.5 ± 30.7 [2650.2, 2770.7]	90.8 ± 0.9 [89.1, 92.5]
Euromonitor “Optimistic” Scenario 1A ^1^	4.9 ± 0.1 [4.7, 5.2] *	1741.7 ± 11.7 [1718.7, 1764.8] *	21.4 ± 0.4 [20.6, 22.2] *	2542.0 ± 31.8 [2479.5, 2604.5] *	82.6 ± 0.8 [81.1, 84.1] *
Euromonitor “Realistic” Scenario 1B ^2^	5.0 ± 0.1 [4.7, 5.2] *	1747.0 ± 11.8 [1723.9, 1770.2] *	21.5 ± 0.4 [20.7, 22.3] *	2543.0 ± 32.2 [2479.8, 2606.3] *	83.8 ± 0.7 [82.3, 85.2] *
CNF “Optimistic” Scenario 2A ^3^	−0.9 ± 0.1 [−1.1, −0.8] *	1703.8 ± 16.1 [1672.1, 1735.5] *	12.8 ± 0.3 [12.2, 13.4] *	909.3 ± 8.9 [891.8, 926.7] *	58.1 ± 0.9 [56.4, 59.8] *
CNF “Realistic” Scenario 2B ^4^	3.7 ± 0.1 [3.5, 3.9] *	2838.6 ± 33.7 [2772.4, 2904.9] *	19.7 ± 0.5 [18.7, 20.6] *	2330.7 ± 39.4 [2253.4, 2408.1] *	85.9 ± 0.8 [84.4, 87.4] *

Results are presented as means ± standard error of the means [95% confidence interval]. * Indicates results significantly differed from the baseline values (*p* < 0.05). ^1^ Consumed items substituted with Euromonitor matches in the same food category with the lowest available Ofcom score or ^2^ a lower Ofcom score. Items with no appropriate match were left as is. ^3^ Consumed items substituted with Canadian Nutrient File (CNF) matches in the same food category with the lowest available or ^4^ next-lowest available Ofcom score. Items with no appropriate match were left as is.

**Table 2:** Ofcom scores and nutrient intakes reported in the Canadian Community Health Survey–Nutrition (CCHS–Nutrition) 2015 PUMFs for baseline data and substitution scenarios, limited to only the foods that were substituted in Scenarios 1A and 1B and could be substituted with a Euromonitor food match (2.9% of total foods consumed) (*n* = 17,503).

	Dietary Ofcom Score	Daily Calories, kcal	Daily Saturated Fat, g	Daily Sodium, mg	Daily Total Sugars, g
Baseline data	10.4 ± 0.2 [10.0, 10.8]	351.7 ± 5.8 [340.2, 363.1]	4.9 ± 0.1 [4.7, 5.1]	573.8 ± 8.9 [556.4, 591.2]	18.1 ± 0.5 [17.0, 19.1]
Euromonitor “Optimistic” Scenario 1A ^1^	6.2 ± 0.2 [5.9, 6.6] *	317.7 ± 6.5 [304.9, 330.5] *	3.7 ± 0.1 [3.5, 4.0] *	428.5 ± 6.8 [415.1, 441.8] *	9.5 ± 0.4 [8.7, 10.4] *
Euromonitor “Realistic” Scenario 1B ^2^	6.8 ± 0.2 [6.3, 7.2] *	323.7 ± 6.2 [311.5, 335.9] *	3.8 ± 0.1 [3.6, 4.1] *	429.6 ± 7.0 [416.0, 443.3] *	10.9 ± 0.3 [10.2, 11.6] *

Results are presented as means ± standard error of the means [95% confidence interval]. * Indicates results significantly differed from the baseline values (*p* < 0.05). ^1^ Consumed items substituted with Euromonitor matches in the same food category with the lowest available Ofcom score or ^2^ a lower Ofcom score.

## Data Availability

The data presented in this study are available on request from the corresponding author because the data are not publicly available.
